# LncRNA *MALAT1* silencing protects against cerebral ischemia-reperfusion injury through miR-145 to regulate AQP4

**DOI:** 10.1186/s12929-020-00635-0

**Published:** 2020-03-06

**Authors:** Hongwei Wang, Xiaoxiao Zheng, Jing Jin, Li Zheng, Ting Guan, Yangfan Huo, Shufen Xie, Ying Wu, Wei Chen

**Affiliations:** 1 Department of anesthesiology, Tongde hospital of Zhejiang Province, Hangzhou, 310012 Zhejiang China; 2 Cancer Institute of Integrated traditional Chinese and Western Medicine, Zhejiang Academy of Traditional Chinese Medicine, Tongde hospital of Zhejiang Province, NO.234, Gucui Road, Hangzhou, 310012 Zhejiang China; 3 Department of Medical Oncology, Tongde hospital of Zhejiang Province, Hangzhou, 310012 Zhejiang China; 4 Department of Brain Surgery,The First Affiliated Hospital, Zhejiang University School of Medicine, Hangzhou, 310003 Zhejiang China

**Keywords:** MALAT1, miR-145, Cerebral ischemia-reperfusion injury, AQP4

## Abstract

**Background:**

The present study aimed to verify whether long noncoding RNA (lncRNA) *MALAT1* is involved in brain tissue damage induced by ischemia-reperfusion injury, and to explore the mechanism by which *MALAT1* regulates aquaporin 4 (AQP4).

**Methods:**

In this study, we established glucose deprivation (OGD)/reoxygenation (RX) astrocyte cell model and middle cerebral artery occlusion (MCAO)/reperfusion mouse model in vitro and in vivo. Then cell counting kit-8 assay, flow cytometry analysis, Triphenyltetrazolium chloride (TTC) staining, and western blotting were used to determine cell viability, cell apoptosis, cerebral infarction volume, and the abundance of AQP4, respectively.

**Results:**

We found that the level of *MALAT1* was significantly upregulated in both the MCAO/reperfusion model and OGD/RX model. Knockdown of MALAT1 increased cell viability and reduced cell apoptosis in MA-C cells, while an AQP4 siRNA combined with a siRNA targeting *MALAT1* could not enhance this effect. Further experiments showed that *MALAT1* positively regulated AQP4 expression via miR-145. The MALAT1 siRNA did not alleviate the exacerbation of damage after miR-145 inhibitor action. However, an miR-145 inhibitor reversed the protection effects of *MALAT1*, indicating that *MALAT1* silencing protects against cerebral ischemia-reperfusion injury through miR-145. TTC staining showed that the infracted area of whole brain was significantly attenuated in treated with sh-MALAT1 group in vivo.

**Conclusion:**

Taken together, our study confirmed that *MALAT1* promotes cerebral ischemia-reperfusion injury by affecting AQP4 expression through competitively binding miR-145, indicating that *MALAT1* might be a new therapeutic target for treatment cerebral ischemic stroke.

## Background

Stroke is one of the most common causes of long-term severe disability and death worldwide [1, 2]. Approximately 80–85% of stroke cases are induced by cerebral ischemia, which is usually caused by embolism or thromboembolism occlusion of the major cerebral aorta [3]. Interventions require recovery of blood flow, resulting in reperfusion injury. Cerebral ischemia-reperfusion (CIR) injury is a pathological process in which nerve damage caused by ischemia and hypoxia is further aggravated following the short-term recovery of blood perfusion [4]. A growing body of evidence suggests that ischemia often involves a range of neurological events, such as, hypoxia, oxidative stress, and an inflammatory response [5], which ultimately lead to acute necrosis, apoptosis, and autophagy in the ischemic brain [6]. Currently, tissue plasminogen activator (tPA) is the only effective method to treat CIR injury [7]. Therefore, it is necessary and urgent to identify novel and effective therapeutic targets, and the underlying molecular mechanism of cerebral ischemia for patients with stroke.

Long noncoding RNAs (lncRNAs) are non-protein coding RNAs of more than 200 nucleotides in length, which participate in various biological processes, such as cell apoptosis, differentiation, angiogenesis, and proliferation [8–10]. Furthermore, lncRNAs also regulate gene expression [11] and are closely associated with various neurological diseases including ischemia stroke [12]. Metastasis-associated lung adenocarcinoma transcript 1 (*MALAT1*), also called non-coding nuclear-enriched abundant transcript 2 *(NEAT2*), was originally identified as being associated with lung cancer metastasis [13, 14]. Extensive evidence revealed that the level of *MALAT1* is upregulated in many cancers and is also associated with tumor initiation, progression, and recurrence [15–17]. *MALAT1* also regulates endothelial cell function and vessel growth [18]. More interestingly, Zhang et al. reported that *MALAT1* was highly expressed during in vitro mimicking of ischemic stroke conditions [19]. A growing number of studies have demonstrated that *MALAT1* is associated with ischemic stroke, and could reduce the number of apoptotic neuronal cells, and inhibit autophagy by regulating microRNA miR-30 in cerebral ischemic stroke [20, 21]. Furthermore, upregulation of *MALAT1* could reduce the protective role of fentanyl in ischemia/reperfusion (I/R) injury by regulating the miR-145/BCL2 interacting protein 3 (BNIP3) pathway [22]. Our previous study demonstrated that overexpression of miR-145 could ameliorate astrocyte cell injury by downregulating aquaporin 4 (AQP4) expression in cerebral ischemic stroke [23]. In the present study, we employed a glucose deprivation (OGD)/reoxygenation (RX) astrocyte cell model and middle cerebral artery occlusion (MCAO)/reperfusion mouse model for in vitro and in vivo study, respectively. Then we investigated the role of *MALAT1* in cerebral I/R injury, and revealed its possible underlying mechanism.

## Methods

### Animals

Six-week-old male C57BL/6 J mice (20–25 g) were purchased from the Experimental Animal Center of Zhejiang University School of Medicine. All mice were housed in an environmentally controlled room under a 12 h light/dark cycle with ad libitum access to food and water. All animal experiments were approved by the First Affiliated Zhejiang Hospital, Zhejiang University of Medical Ethics Committee and the Medical Faculty Ethics Committee of the First Affiliated Zhejiang Hospital, Zhejiang University in accordance with the National Institutes of Health Guide for Care and Use of Laboratory Animals (NIH Publications, No. 8023, revised 1978).

### Primary astrocyte culture

Primary astrocytes were prepared from a post-natal day (PND) 7 cerebellum from C57BL/6 J mice, and the protocol used was described previously [24]. Briefly, the tissue was incubated for 15 min in 0.065% trypsin at 37 °C and then centrifuged 1500 g for 5 min. The cell pellet was treated with 0.004% DNase I for 7 min at 37 °C. After centrifugation 1500 g for 5 min, the cell pellet was gently resuspended in a small volume of tissue growth medium (Dulbecco’s modified Eagle’s medium containing 10% FBS) and plated in the same medium at a density of 2 × 10^5^ cells/cm^2^ in 35-mm Corning culture dishes precoated with 100 μg/ml poly-D-lysine. The cells were cultured in poly-L-lysine–coated 35-mm dishes with DMEM containing 10% FBS at 37 °C in 5% CO_2_ in a humidified.

### Astrocyte cell OGD/RX model

The astrocyte cell OGD/RX model was established in accordance with the methods as previously described [25]. Briefly, the cells were transferred to glucose-free DMEM and cultivated in a humidified incubator with 95% N_2_ and 5% O_2_ at 37 °C for 6 h. For reperfusion, the medium was replaced with high-glucose DMEM and incubated in a normoxic incubator for an additional 24 h or 48 h.

### Middle cerebral artery occlusion (MCAO)/reperfusion model

The Middle Cerebral Artery Occlusion (MCAO) procedure was described in our previous study [26]. In brief, mice were anesthetized with 4% chloral hydrate (Sigma, St. Louis, MO, USA) and a 6–0 silicone-coated nylon monofilament (Doccol Corp., Redlands, CA, USA) was inserted into the left common carotid artery to occlude the MCA origin. After 1 h, the suture was removed. The blank groups prepared the filaments and inserted into the left common carotid artery. The mice were then anesthetized and decapitated to obtain the brain. C57BL/6 J mice were randomly grouped as follows (*n* = 5 for each group): NC group (Threading without occlusion, followed by persistent perfusion), MCAO 24 h (1 h of ischemia and 24 h of reperfusion), MCAO 48 h (1 h of ischemia and 48 h of reperfusion), shMALAT1 (short hairpin RNA targeting MALAT1) + MCAO 48 h (0.5 mg/kg MALAT1 shRNA, via intracerebroventricular injection before MCAO). MALAT1 shRNA or sh-NC was injected into the right cerebral ventricle of mice. One day post-injection, MCAO operation was established.

### Measurement of the cerebral infarction area

Triphenyltetrazolium chloride (TTC) staining was used to determine cerebral infarction area. After 24 h of reperfusion, 1 mm-thick coronal sections of the brain were immersed in a 2% TTC solution at 37 °C for 30 min. The total area of each brain section and the infarcted region were quantified using Leica image software DMI6000B (Leica Microsystems, Wetzlar, Germany). The infarct volume was determined as follows: (infarcted area /total brain area) × 100%.

### siRNA transfection

The miR-145 mimic, miR-145 inhibitor, or MALAT1 siRNA were synthesized by GenePharma (Shanghai, China). MA-C cells, which were obtained from ATCC (Manassas, VA, USA), were transfected using Lipofectamine 2000 (Invitrogen; Thermo Fisher Scientific, Waltham, MA, USA) according to the manufactures’ instructions. The oligonucleotide and MALAT1 siRNA sequences used were as follows:

hsa-miR-145 mimics:

Forward 5′- GUCCAGUUUUCCCAGGAAUCCCU − 3′

Reverse 5′- GGAUUCCUGGGAAAACUGGACUU − 3′

hsa-miR-145 inhibitor:

5′- AGGGAUUCCUGGGAAAACUGGAC -3′

MALAT1 siRNA:

MALAT1–1-Homo-209:

Forward 5′- GGUGGUGGUAUUUAGAUAATT − 3′

Reverse 5′- UUAUCUAAAUACCACCACCTT -3′;

MALAT1–2-Homo-1790:

Forward 5′- GCGUCAUUUAAAGCCUAGUTT − 3′;

Reverse 5′- ACUAGGCUUUAAAUGACGCTT -3′;

MALAT1–3-Homo-4443:

Forward 5′- GGGCUGACAUUAACUACAATT − 3′

Reverse 5′- UUGUAGUUAAUGUCAGCCCTT − 3′.

### Western blot analysis

Protein samples (40 μg/lane) were separated using 10% SDS-PAGE, and then transferred to a polyvinylidene difluoride (PVDF) membrane (Millipore, Billerica, MA, USA). After blocking with Tris-buffered saline (TBS) and 0.1% Tween-20 (TBS/T) containing 5% bovine serum albumin (BSA), the membranes were incubated with primary antibodies against AQP4 (Abcam, Cambridge, MA USA) diluted 1:1000 in TBS/T overnight at 4 °C. The membranes were washed and incubated with a horseradish peroxidase-labeled secondary antibody diluted 1:2000 at room temperature for 2 h. Subsequently, the protein bands were assessed using chemiluminescence (GE Healthcare, Piscataway, NJ, USA). The immunoreactive protein bands were quantified using Image Lab 5.0 (Bio-Rad, Hercules, CA, USA) and glyceraldehyde-3-phosphate dehydrogenase (GAPDH) was used as an internal control.

### Quantitative real-time reverse transcription PCR (qPCR)

Total RNA was isolated using the TRIzol reagent (Invitrogen; Thermo Fisher Scientific, Inc.). cDNA was synthesized using a PrimeScript RT Reagent Kit (Takara, Shiga, Japan). After the RT reaction, 1 μl of cDNA was used for subsequent qPCR with SYBR Green dye (Takara) using a 7500 Real-Time PCR System (Applied Biosystems; Thermo Fisher Scientific, Inc.). The PCR conditions were as follows: 40 cycles of 95 °C for 30 s, 60 °C for 34 s, and 72 °C for 30 s. U6 and β-actin were used as internal controls, and the relative expression levels were calculated using the 2^-ΔΔCt^ method [27]. All reactions were performed in triplicate. The primer sequences were as follows:

MALAT1:

Forward 5′- TGTGACGCGACTGGAGTATG − 3′

Reverse 5′- CAAAGGGACTCGGCTCCAAT -3′;

AQP4:

Forward 5′-GACAGACCCACAGCAAGG-3′

Reverse 5′- GCAAAGGGAGATGAGAACC-3′;

miR-145:

5′-GTCCAGTTTTCCCAGGAATCCCT-3′;

U6:

Forward 5′-CTCGCTTCGGCAGCACA-3′,

Reverse 5′-AACGCTTCACGAATTTGCGT-3′;

β-Actin:

Forward 5′- GACTTAGTTGCGTTACACCCTT-3′,

Reverse 5′- TTTTGACCTTGCCACTTCCA-3′

### Cell Calcein-AM/PI staining assay

Cell viability was measured using a calcein-acetoxymethyl (AM)/propidium iodide (PI) double staining kit (Dojindo, Kumamoto, Japan). In brief, cells were fixed with 70% ethanol for 15 min. They were then stained with calcein-AM solution (10 μmol/l) for 15 min. They were then washed with phosphate-buffered saline (PBS) buffer and stained with PI (10 μmol/l) for 15 min. The optical density at 490 nm and 535 nm were determined to determine healthy cells and dead cells, respectively.

### Lactate dehydrogenase (LDH) analysis

The level of lactate dehydrogenase (LDH) was determined using a commercial Cytotoxicity LDH Assay Kit-WST according to the manufacturer’s instructions (Dojindo, Kumamoto, Japan). In brief, MA-C cells were cultured in 6-well plates, following transfection and OGD/RX treatment, then collected, re-suspended, couted and seeded in a 96-well plate at 37 °C in 5% CO_2_. Add 20 μl of the Lysis Buffer to each well of the high control. Incubate the plate at 37 °C for 30 min in a CO_2_ incubator. 100 μl working solution was added to each well. Protect the plate from light and incubate it at room temperature for 30 min. At last, 50 μl stop solution was added to each well. The level of LDH was measured the absorbance at 490 nm by a microplate reader.

### Bederson score

Normal mice exhibited a head lift and the two front paws extended to the tabletop when the mouse tail was raised to 5 cm above the table top. The brain injury mice displayed buckling of the contralateral forelimbs. The postural changes from mild flexion, elbow extension, shoulder abduction, severe wrist and elbow flexion, and shoulder internal rotation abduction were observed. The Bederson score was divided into four grades according to the posture reflex and shoulder lateral thrust test results of the mice. 0 point: no neurological deficit symptoms were observed; 1 point: buckling of the contralateral forelimbs of the infarct hemisphere when the tail was suspended, but not with other abnormalities; 2 points: when the tail is suspended, the contralateral forelimb of the infarct hemisphere is flexed, and the opposite side of the infarct hemisphere is pushed by hand, and its resistance is reduced, but it does not rotate when freely moving; 3 points: the same behavior with 2 points and when it is free to move, turn to the side of the squat.

### TdT-mediated biotin-16-dUTP nick-end labeling (TUNEL) assay

A TUNEL Apoptosis Assay kit (Roche, Basel, Switzerland) was used to detect the apoptotic cells according to manufactures’ instructions. Briefly, paraffin-embedded sections were deparaffinized and hydrated in a graded ethanol series and then digested with trypsin for 40 min at room temperature. The tissue sections were then incubated with TUNEL reaction buffer in a 37 °C humidified atmosphere for 60 min, and then washed with PBS. TUNEL-positive cells and normal cells in each group were counted under a light microscope at 200× magnification (Olympus, Tokyo, Japan).

### Flow cytometry analysis

The number of apoptotic cells in the different treatment groups was examined using Flow cytometry analysis. The cells were stained with Annexin V/fluorescein isothiocyanate (FITC) kit (BD Biosciences, Franklin Lakes, NJ, USA) according to the manufacturer’s instructions. Briefly, MA-C cells were cultured in 6-well plates, following OGD/RX treatment and transfection. The cells were collected,100 μl Binding Buffer and 5 μl fluorescein isothiocyanate (FITC)-labeled Annexin V (20 μg/ml) were added and incubated in the dark at room temperature for 15 min. Then, 5 μl propidium iodide (PI; 50 μg/ml) was added and incubated in the dark for 5 min. Thereafter, 400 μl of Binding Buffer was added and immediately subjected to FACScan for quantitative detection by flow cytometry (within 1 h).

### Immunofluorescence

Astrocyte cells were identified using immunofluorescence. Cells were washed with cold PBS, fixed in 4% paraformaldehyde for 15 min, blocked with 5% BSA at 37 °C for 30 min, and incubated with anti-glial fibrillary acidic protein (GFAP) antibodies (1:100; Abcam) overnight at 4 °C. The cells were washed and incubated with secondary antibodies (1:100; Abcam) for 2 h at 37 °C. Nuclei were stained using 2-(4-amidinophenyl)-1H-indole-6-carboxamidine (DAPI; (Sigma) for 2 min at 37 °C. The cells were washed and observed under an inverted fluorescence microscope (Olympus, Tokyo, Japan).

### Statistical analysis

All experimental values are expressed as the mean ± SD. We used GraphPad Prism 6.0 software (GraphPad Software, San Diego, CA, USA) to perform the statistical analysis. Statistical analysis was performed using a *t*-test for the comparison of two conditions. A one-way analysis of variance (ANOVA) with a Bonferroni post-test was used for multiple comparisons. A *P* value of < 0.05 was considered statistically significant.

## Results

### Inhibition of MALAT1 could protect against astrocyte cell injury caused by ischemia- reperfusion

**Fig. 1:**
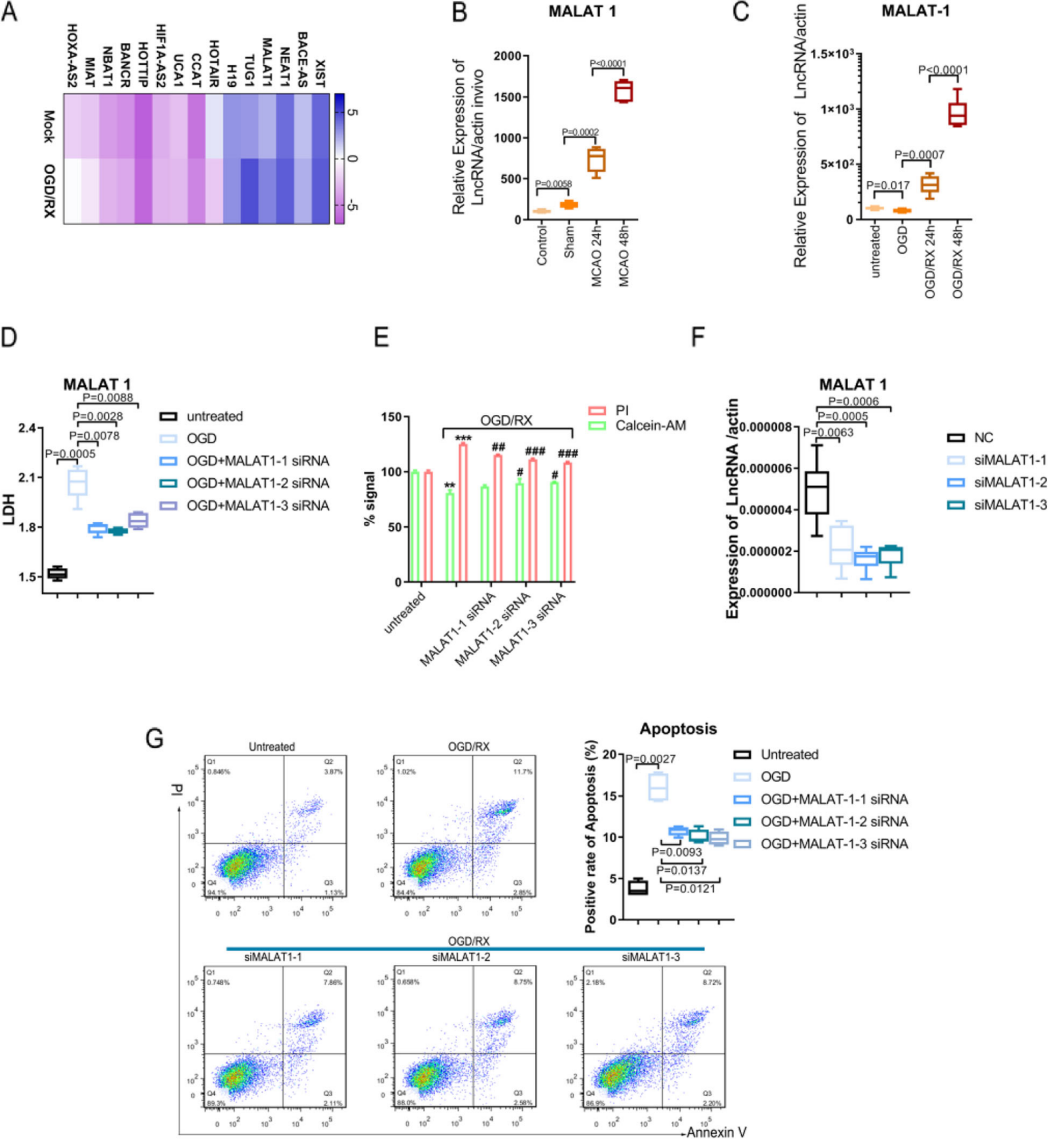
Inhibition of *MALAT1* could protect astrocytes against the cell injury caused by ischemia- reperfusion. **a**. QPCR determining of the level of different LncRNAs under the OGD/RX conditions or Mock. **b**, **c**. The level of MALAT1 was determined by qPCR under different conditions. **d**. Transfected with the *MALAT1* siRNA in OGD conditions decreased the LDH activity. **e**. Cell Calcein-AM/PI staining assay to determine cell viability. **P **<** 0.01,***P < 0.001 vs untreated; #*P* < 0.05,##*P* < 0.01,###*P* < 0.001 vs OGD/RX. **f**. QPCR examination of the interference efficiency of si-MALAT1. **g**. Flow cytometry analysis showing that compared with OGD/RX, the reduction of cell apoptosis following MALTA1 interference in OGD/RX conditions

To search appropriate LncRNAs, we used qPCR to detect the level of lncRNAs under oxygen and glucose deprivation (OGD)/reoxygenation (RX) or Mock condition, which showed that MALAT1 was significantly upregulated under OGD/RX conditions (Fig. 1a). We established MCAO mouse model and OGD/RX cell model and then we examined the level of MALAT1 in vivo and in vitro. We found that the expression of MALAT1 was upregulated in MCAO mice at 24 and 48 h compared with sham group and control group (Fig. 1b). *MALAT1* expression was also significantly increased with OGD/RX for 24 h and 48 h in MA-C cells compared to the OGD only group (Fig. 1c). To observe the effect of *MALAT1* on MA-C cells, a LDH assay showed that knockdown of *MALAT1* could attenuate the increase in OGD-induced LDH release from cells (Fig. 1d). Cell calcein-AM/PI staining experiment was used to determine the percentage of live cells in which calcein-AM stains live cells and PI stains dead cells. The results showed that the percentage of live cells was significantly increased in cells treated with the *MALAT1* siRNA under OGD/RX conditions (Fig. 1e). The transfection efficiency of *MALAT1* siRNA was determined using qPCR (Fig. 1f). Furthermore, flow cytometry was used to determine apoptosis cells, and we found that the number of apoptotic cells was significantly reduced after lncRNA *MALAT1* siRNA intervention (Fig. 1g).

### AQP4 mediates the effect of MALAT1 on the damage to astrocyte cells

**Fig. 2:**
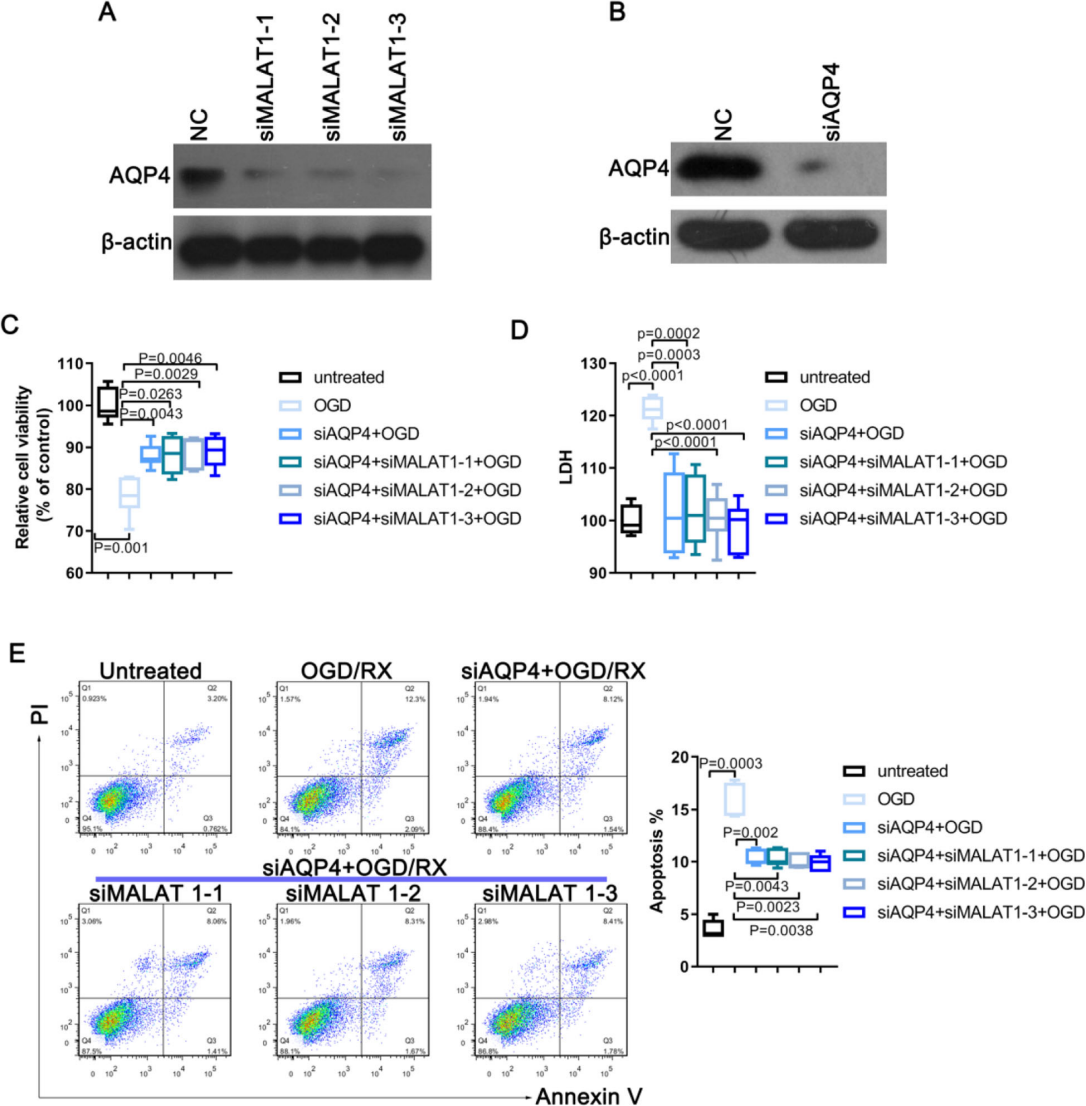
AQP4 mediate the damage of MALAT1 on astrocyte cell. **a**. Western blot showing the level of AQP4 following transfection with or without siMALAT-1, siMALAT-2, siMALAT-3. **b**. Western blot analysis of AQP4 levels after transfection with or without AQP4 siRNA. **c**. Cell viability induced by different groups of OGD/RX was examined by CCK-8 assay. **d**. LDH release was determined in different treatment groups using LDH assay. **e**. The number of apoptotic cells in different groups were measured using Flow cytometry

Our research team has been studying the role of AQP4 at the early stages of stroke brain tissue damage [23]. We also revealed that AQP4 in astrocytes may promote ischemia reperfusion injury (Figure S1). Glial fibrillary acidic protein (GFAP), the hallmark intermediate filament protein in astrocytes, a main type of glial cells in the central nervous system (CNS). GFAP immunofluorescence staining was used for identifying primary astrocytes purity (Figure S1 A). Western Blot and qPCR showed that AQP4 expression is significantly increased after OGD/RX treatment (Figure S1 B-C). To investigate the physiological roles of AQP4 in astrocytes, cells were transfected with AQP4 siRNA to knockdown AQP4 levels (Figure S1 D). Calcein-AM/PI staining were performed to examine the effect of AQP4 on cell viability of astrocytes upon OGD/RX treatment. Intriguingly, knockdown of AQP4 significantly heightened cell viability against OGD/RX treatment (Figure S1 B). Additionally, reduced AQP4 levels resulted in a decrease of cytotoxicity by LDH assay(Figure S1 E). Consistently, reduced apoptosis was determined by flow cytometric analysis with AnnexinV/PI staining in astrocytes with AQP4 siRNA upon OGD/RX treatment (Figure S1 F). As a result, we found that lncRNA MALAT1 could regulate the expression of AQP4, and AQP4 mediates the damage of MALAT1 on MA-C cells. Considering the important role of AQP4 in stroke reperfusion injury, we speculated whether LncRNAs play an important role in regulating AQP4. The level of AQP4 was detected before and after *MALAT1* siRNA interference, and the results showed that the level of AQP4 was reduced after knockdown of *MALAT1* (Fig. 2a). After *AQP4* was successfully knocked down in MA-C cells using an siRNA, the OGD/RX model was established, and the model cells treated the *MALAT1* siRNA, or not treated to observe whether the cells were damaged in the presence or absence of *MALAT1*. The efficiency of *AQP4* siRNA silencing was determined using western blotting (Fig. 2b). The LDH and CCK-8 results showed that after AQP4 silencing, cell damage was alleviated, while combined with MALAT1 siRNA, we found that MALAT1 siRNA did not change the effect of AQP4 on the reduction of brain injury after OGD-RX induction (Fig. 2c, d). In addition, to verify whether MALAT1 play a protective effect through regulating AQP4 after OGD/RX, MA-C cells were transfected with AQP4 plasmid to overexpress AQP4 (Figure S2 C), along with or without MALAT1 siRNA upon OGD/RX treatment, respectively. Impressively, the damage effects of OGD/RX on cell viability and cytotoxicity were robustly amplified by the transfection of AQP4 plasmid, while the protective effects of siMALAT1 disappeared when cells were co-transfected with AQP4 plasmid and MALAT1 siRNA (Figure S2 A-B). These findings demonstrated that MALAT1 promoted astrocytes damage through AQP4 upon OGD/RX treatment. Flow cytometry analysis showed that after A*QP4* silencing, *MALAT1* did not further reduce apoptosis, again demonstrated that *MALAT1* acted through AQP4 (Fig. 2e). These results demonstrated that lncRNA *MALAT1* could regulate the expression of AQP4, and that AQP4 mediates the effects of *MALAT1* on the damage to astrocytes.

### MALAT1 positively modulates AQP4 through miR-145 expression

**Fig. 3:**
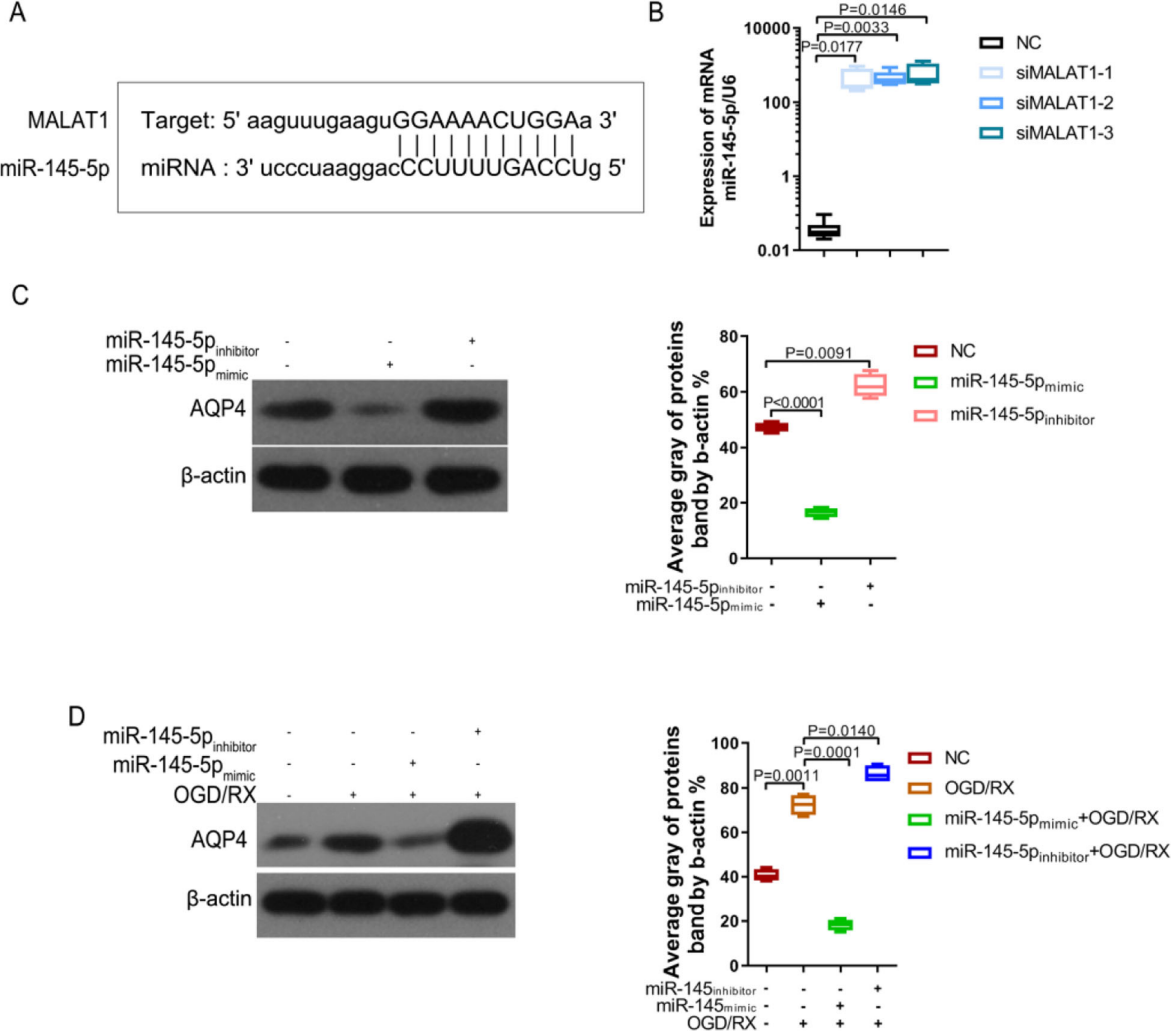
MALAT1 positively modulated AQP4 through miR-145 expression. **a**. StarBase software prediction of the relationship between MALAT1 and miR-145. **b**. MiR-145-5p level was examined using qPCR. **c**. Western blotting analysis of AQP4 expression following transfection with miR-145-5p mimic, inhibitor, or NC. **d**. Western blotting analysis of AQP4 levels after transfection with or without miR-145-5p mimic or inhibitor, with or without OGD/RX conditions

LncRNAs participate in the regulation of biological activity by acting as competing endogenous RNAs (ceRNAs) for miRNAs [28]. To explore the underlying mechanism of *MALAT1* regulating AQP4 in cerebral ischemia-reperfusion injury, we used starBase (http://starbase.sysu.edu.cn/) to screen miRNAs that have complementary base pairing with *MALAT1*. Interestingly, miR-145 was identified as a potential target of *MALAT1* (Fig. 3a). QPCR detected the changes in miR-145 expression with or without MALAT1 siRNA (siMALAT1–1, siMALAT1–2 and siMALAT1–3) transfection, showing that the level of miR-145 was significantly upregulated following knockdown of *MALAT1* (Fig. 3b). Bioinformatics analysis using Targetscan (http://www.targetscan.org/) indicated that miR-145 binds to the 3′ UTR of the *AQP4* mRNA. An miR-145 mimic significantly downregulated AQP4 levels, while an miR-145 inhibitor significantly upregulated AQP4 levels in the astrocytes. Furthermore, in the OGD/RX model, the miR-145 mimic could downregulate the increase of AQP4 caused by OGD/RX, while the miR-145 inhibitor could upregulate AQP4 level (Fig. 3c-d), which was consistent with the results of our previous study [23].

### MiR-145 mediates the effect of MALAT1 on damage to astrocyte cells

**Fig. 4:**
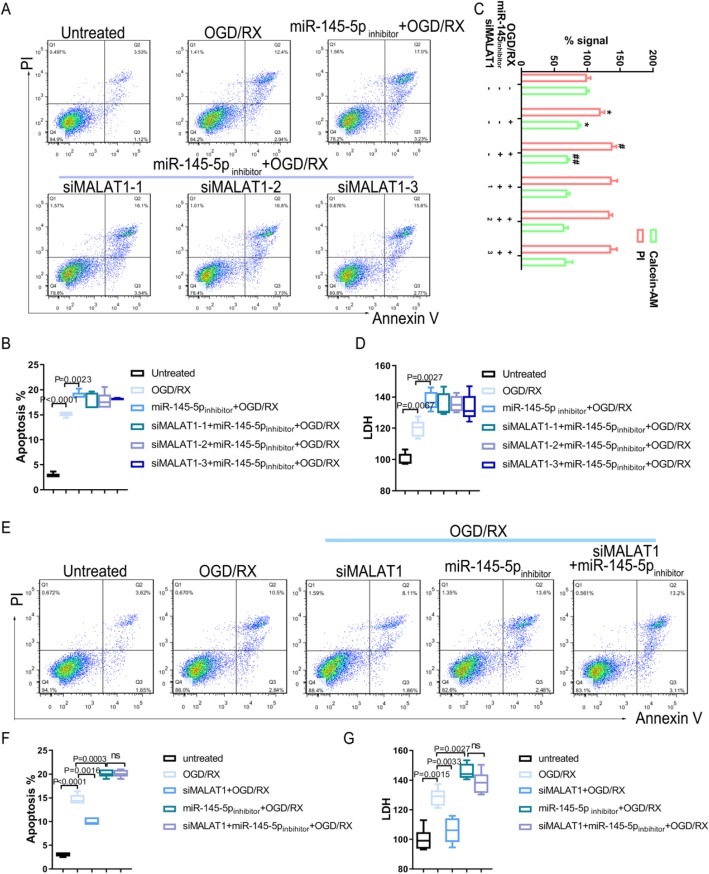
MiR-145 mediates the effect of MALAT1 on the damage to astrocyte cells. **a**-**b**. Flow cytometry analysis of apoptotic cells. **c**. Cell Calcein-AM/PI staining assay to determine cell viability. **d**. LDH assay analysis to determine LDH release in different groups. **e**-**f**. Flow cytometry detection of cell apoptosis after treatment with siMALAT1 alone, miR-145 inhibitor alone, or siMALAT1 plus miR-145 inhibitor in OGD/RX conditions, and Control. **g**. LDH assay analysis was used to detect the LDH activity in the different groups

To further verify whether miR-145 was a target of *MALATA1* mediating its regulation of AQP4 on OGD/RX damage to astrocyte cells, MA-C cells were pretreated with the miR-145 inhibitor along with or without the *MALATA1* siRNA upon OGD/RX treatment, cell viability, cell apoptosis and cytotoxicity were detected via Calcein-AM/PI staining, flow cytometric analysis and LDH assay, respectively. Flow cytometry detection of apoptotic cells revealed that after treatment with the miR-145 inhibitor, the number of apoptotic cells increased, while treatment with the *MALAT1* siRNA did not influence the effect of the miR-145 inhibitor (Fig. 4a-b). Furthermore, the percentage of dead cells stained by PI and cytotoxicity LDH release increased significantly by treatment with the miR-145 inhibitor under OGD/RX conditions; however, knockdown of *MALAT1* did not alleviate the astrocytes injury (Fig. 4c-d). Next, we found that *MALAT1* siRNA interference could reduce cell apoptosis and cytotoxicity LDH release which were induced by OGD/RX treatment. Although knockdown of *MALAT1* could protect MA-C cells from OGD/RX damage, miR-145 inhibitor could reverse the influence of *MALAT1* siRNA (Fig. 4e-g). These results demonstrated that the effects of the *MALAT1* siRNA act via upregulating miR-145levels.

### MALAT1 is involved in the regulation of stroke injury by upregulating miR-145 in vivo

**Fig. 5:**
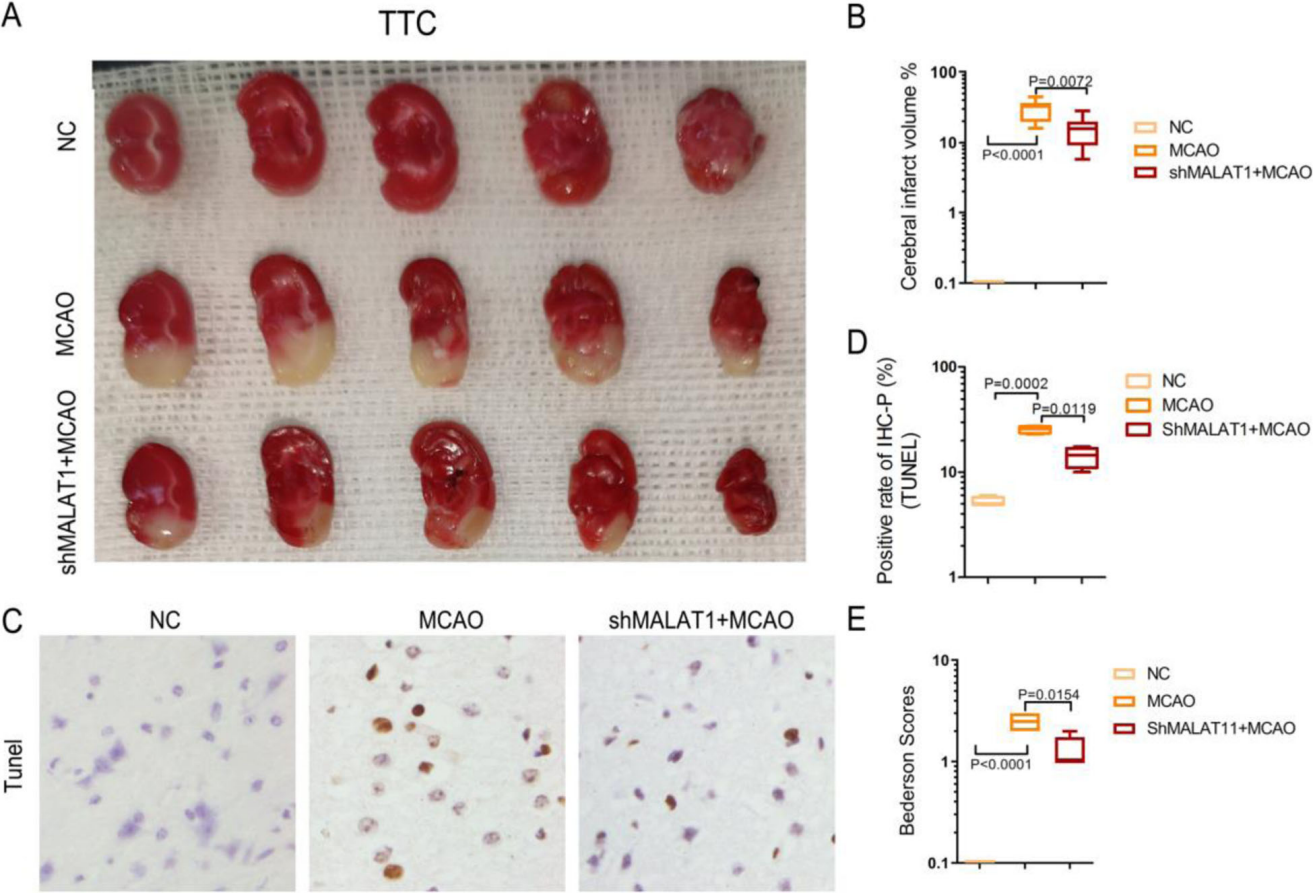
*MALAT1* is involved in the regulation of stroke injury by upregulating miR-145 in vivo*.***a**-**b**. TTC staining was used to evaluate the infarct volume induced by MCAO, and MALAT1 shRNA injection in MCAO. **c**-**d**. Tunel assays were performed to determine the number of apoptotic cells in the different groups (NC, MCAO, shMALAT1 + MCAO) **e**. The Bederson Scores of the mice treated with or without shMALTA1 after MCAO to evaluate their neurological behavior

In vivo experiments confirmed that lncRNA *MALAT1* was involved in the regulation of stroke damage. The brain infarct area was determined via a 2,3,5-triphenyltetrazolium chloride (TTC) stain assay at 24 h of reperfusion after MACO surgery. The infarct volume in the Con shRNA-injected MACO mice was significantly increased compared with the NC group, but this increase was inhibited by *MALAT1* shRNA injection (Fig. 5a-b). To further verify this observation, TUNEL apoptosis detection assay was utilized. We found that TUNEL positive cells in brain infarct area slices were significantly increased in the Con shRNA-injected MACO group compared with NC group, while shMALAT1 could ameliorate cell apoptosis (Fig. 5c-d). Neurological deficit was evaluated at 24 h after MCAO. Neurological deficit assessment was performed by a researcher blinded to the experimental groups. Neurological function measurement was determined using Bederson scores test. The Bederson score was graded on a scale of 0 to 3 (normal score, 0; maximum score, 3), so the score of NC group was 0. Consistent with the brain damage findings, the Bederson scores were significantly decreased in *MALAT1* shRNA-injected mice compared with those in the Con shRNA-injected mice after 24 h of reperfusion (Fig. 5e).

## Discussion

The development of effective treatment is critical for ischemia stroke, because it causes high mortality and disability worldwide. LncRNAs are a newly discovered class of regulators in the cerebral vascular endothelium after ischemic injury, such as stroke [29]. In recent years, non-coding RNAs have emerged as novel targets to treat ischemia stroke [30, 31]. For example, inhibition of *LncCHRF* reduced ischemic injury by regulating the miR-126/SOX6 axis [32]. Knockdown of LncRNA *AK038897* could protect against cerebral ischemia/reperfusion injury by regulating miR-26a-5 targeting of DAPK1 [33]. Thus, lncRNAs have great potential as new therapeutic targets in the progression of cerebral ischemia/reperfusion injury. Resent study had demonstrated that MALAT1 also play an important role in cerebral ischemia/reperfusion injury [34]. For instance, silencing of MALAT1 could inhibit OGD-R-induced apoptosis [35]. Down-regulation of MALAT1 suppressed ischemic injury and autophagy in vitro and in vivo [21]. Furthermore, MALAT1 silencing presented with larger brain infarct size, worse neurological scores, and reduced sensorimotor functions post-MCAO [20].In the present study, we demonstrated that inhibition of *MALAT1* had a protective effect against cerebral ischemia/reperfusion injury by regulating miR-145/AQP4 expression. We found that *MALAT1* levels were significantly upregulated in the MCAO mouse model at 24 and 48 h compared with sham group in vivo. Furthermore, the longer the MCAO time, the higher the *MALAT1* expression. In vitro, *MALAT1* expression was also significantly increased after OGD/RX in MA-C cells compared with the OGD group. These results were consistent with Guo et al reported [21]. Moreover, under OGD/RX conditions, knockdown of *MALAT1* reduced cell apoptosis. Compared with the OGD/RX group, the percentage of live cells increased after transfection with the *MALAT1* siRNA under OGD/RX conditions, suggesting that MALAT1 siRNA might have a protective effect in cerebral ischemia/reperfusion injury, which was consistent with the results reported in a previous study [21].

Aquaporin 4 (AQP4), a small monomer, is a hydrophobic transmembrane protein with cytosolic amino and carboxy termini. AQP4 is abundantly expressed in astrocytes and is involved in the occurrence of brain edema following intracerebral hemorrhage [36, 37]. Accumulating evidence suggests that AQP4 serves as a potential therapeutic target for ischemic injury [26, 38, 39]. In this study, we determined that the expression of AQP4 was increased after OGD/RX, and that AQP4 siRNA interference could reduce cell apoptosis, which was consistent with a previous report [26]. We also found that *MALAT1* could regulate the expression of AQP4. Furthermore, transfection with the AQP4 siRNA combined with the *MALAT1* siRNA under OGD/RX conditions had no significant effect on cell viability, LDH release, and apoptotic cells compared with OGD/RX alone group. These results revealed that AQP4 mediates the effect of *MALAT1* on the damage to astrocytes cells.

MicroRNAs (miRNAs), short non-coding RNAs of about 18~21 nucleotides in length, can regulate mRNA translation by targeting their 3′ untranslated region (3′-UTR) [40]. More than 20% of miRNAs are abnormally expressed in the ischemic brain, suggesting that miRNAs are involved in the pathogenesis and development of ischemic stroke [41, 42]. Overexpression of miR-424 could reduce ischemic brain injury through suppressing microglia activation [43]. Increasing miR-224-3p expression could attenuate cerebral ischemia/reperfusion injury by decreasing the expression of FAK family-interacting protein (FIP200) [44]. Our previous study demonstrated that upregulation of miR-145 could reduce astrocyte injury by decreasing the expression of AQP4 [23]. The present study identified *MALAT1* as a regulator of cerebral ischemia/reperfusion injury by regulating miR-145 to target AQP4. Ren et al. and Xiang et al. had indicated that *MALAT1* and miR-145 could bind directly to each other, which was similar to our prediction [45, 46]. Knockdown of *MALAT1* significantly upregulated the level of miR-145, and an miR-145 inhibitor could increase the expression of AQP4. Moreover, when we transfected cells with the miR-145 inhibitor under OGD/RX condition, the astrocytes cells were damaged more seriously compared with the OGD/RX group; however, treatment with the *MALAT1* siRNA abrogated the protective effect of *MALAT1* following transfection with the miR-145 inhibitor under OGD/RX conditions, and the miR-145 inhibitor could reverse the protection effects of *MALAT1*. In vivo, we also found that inhibition of *MALAT1* in the MCAO model could reduce the infarct area of the whole brain and the number of apoptotic cells.

## Conclusion

In conclusion, the results of the present study demonstrated that inhibition of *MALAT1* could protect against cerebral ischemia/reperfusion injury by downregulating AQP4 levels via miR-145. These findings might provide novel therapeutic targets for the treatment of cerebral ischemia stroke.

## Supplementary information

**Additional file 1 : Figure S1.** AQP4 could promote injury to astrocyte cells caused by ischemia reperfusion. **Figure S2.** A. Cell viability was determined by CCK-8 assay in different treatment groups (untreated, OGD, AQP4 plasmid+OGD, AQP4 plasmid+*MALAT1* siRNA+OGD). B. MA-C cells were transfected with AQP4 plasmid, or combined with *MALAT1* siRNA. OGD/RX condition was employed and the level of LDH was detected by LDH assay. C. MA-C cells were transfected with AQP4 plasmid. AQP4 protein expression were assessed using Western Blot.

## Data Availability

All data generated or analyzed during this study are included in this published article.

## Abbreviations

AQP4: Aquaporin 4
BNIP3: miR-145/BCL2 interacting protein 3
CIR: Cerebral ischemia-reperfusion
I/R: Ischemia/reperfusion
lncRNAs: Long noncoding RNAs
*MALAT1*: Metastasis-associated lung adenocarcinoma transcript 1
MCAO: Middle Cerebral Artery Occlusion
*NEAT2*: Non-coding nuclear-enriched abundant transcript 2
tPA: Tissue plasminogen activator
TTC: Triphenyltetrazolium chloride
