# Supplementary material for: LncRNA MALAT1 silencing protects against cerebral ischemia-reperfusion injury through miR-145 to regulate AQP4
Source: J Biomed Sci. 2020 Mar 6;27:40. doi: 10.1186/s12929-020-00635-0 (PMC7059719; doi:10.1186/s12929-020-00635-0)
Supplement: Supplementary file 1 — Additional file 1 : Figure S1. AQP4 could promote injury to astrocyte cells caused by ischemia reperfusion. Figure S2. A. Cell viability was determined by CCK-8 assay in different treatment groups (untreated, OGD, AQP4 plasmid+OGD, AQP4 plasmid+MALAT1 siRNA+OGD). B. MA-C cells were transfected with AQP4 plasmid, or combined with MALAT1 siRNA. OGD/RX condition was employed and the level of LDH was detected by LDH assay. C. MA-C cells were transfected with AQP4 plasmid. AQP4 protein expression were assessed using Western Blot. [file 12929_2020_635_MOESM1_ESM.docx]

**Supplementary data**


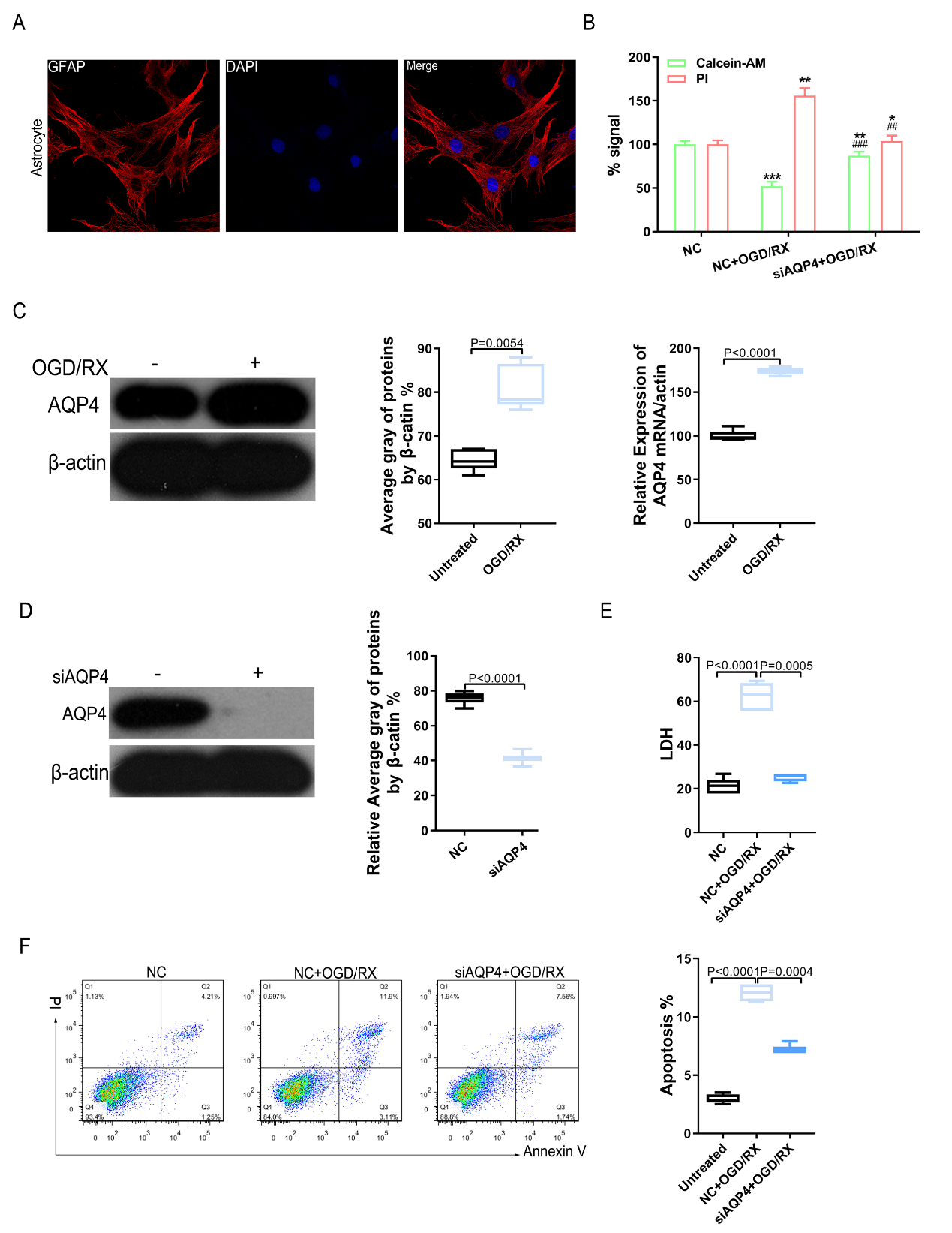


**Figure S1. AQP4 could promote injury to astrocyte cells caused by ischemia reperfusion**

A. Primary astrocytes were identified by GFAP immunostaining (Red). B. Calcein-AM/PI assay was used to observe healthy cells and dead cells in NC group, NC+OGD/RX group, and AQP4 siRNA+OGD/RX group. *P < 0.05,**P < 0.01, ***P < 0.001 *vs*. NC; ##P < 0.01, ###P < 0.001 *vs*. NC+OGD/RX. C. Western blotting was performed to detect the protein level of AQP4 after OGD/RX or NC. D. The interference efficiency of siAQP4 was determined by western blotting. E. The level of LDH was determined by LDH assay analysis.


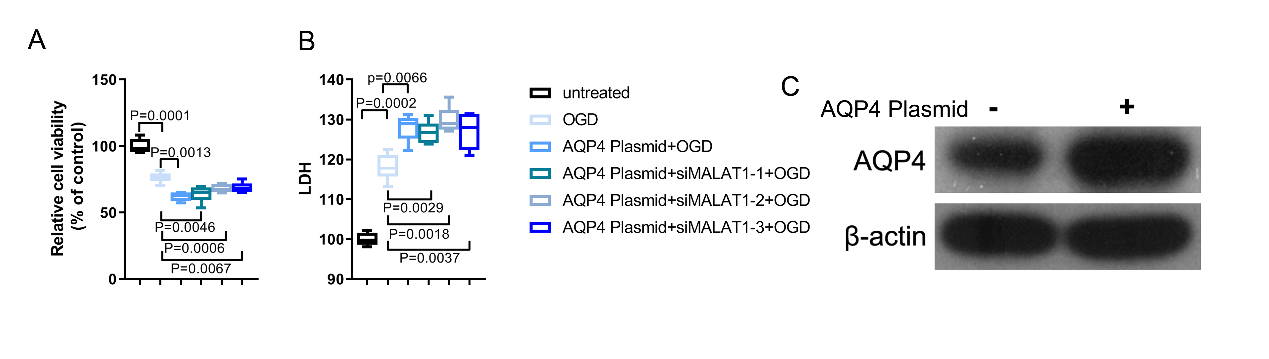


**Figure S2**

**A. Cell viability was determined by CCK-8 assay in different treatment groups (untreated, OGD, AQP4 plasmid+OGD, AQP4 plasmid+*MALAT1* siRNA+OGD). B. MA-C cells were transfected with AQP4 plasmid, or combined with *MALAT1* siRNA. OGD/RX condition was employed and the level of LDH was detected by LDH assay. C. MA-C cells were transfected with AQP4 plasmid. AQP4 protein expression were assessed using Western Blot.**
